# Intra-individual Variations in Voice Variables Among Individuals With and Without Parkinson’s Disease

**DOI:** 10.7759/cureus.81398

**Published:** 2025-03-29

**Authors:** Paula Abola, Mitchell Wolden

**Affiliations:** 1 Clinical Research, University of Jamestown, Fargo, USA; 2 Physical Therapy Program, University of Jamestown, Fargo, USA

**Keywords:** jitter, parkinson' s disease, speech disorders, voice changes, voice disorder

## Abstract

Introduction

Voice changes affect 70 to 90% of individuals with Parkinson’s disease (PD). Voice and speech changes in individuals with PD include increased shimmer, increased jitter, reduced harmonics-to-noise ratio (HNR), and changes in fundamental frequency. Studies have identified inter-individual variations in voice variables as PD progresses. However, intra-individual variations in voice variables have not been studied extensively. When individuals without PD experience stress or nervousness, their jitter and shimmer may also increase. Therefore, the purpose of our study was to compare the mean intra-individual variations in voice variables for individuals with PD and those without PD to determine whether the mean intra-individual variations differ between the two groups.

Methodology

We utilized the “Oxford Parkinson’s Disease Detection Dataset,” which consists of various biomedical voice measurements, and each individual’s voice was measured six or seven times. The changes between each voice measurement for each individual were calculated. Independent samples t-tests were performed to determine significant differences in intra-individual variations in voice variables between individuals with PD and those without PD for all voice variables.

Results

The independent samples t-tests revealed no statistically significant differences in intra-individual variations between individuals with (n = 24) and without PD (n = 8) for any of the voice variables. For vocal fundamental frequency variables, the mean differences ranged from -1.98e5 (Flo) to 2.58e5 (Fhi). For jitter variables, the mean differences ranged from 1.05e-6 (Jitter(Abs)) to 5.32e-4 (DDP). For shimmer variables, the mean differences ranged from 2.11e-4 (APQ5) to 7.07e2 (Shimmer(dB)). For other variables, the mean differences ranged from -2.76e6 (HNR) to 6.02e4 (spread1).

Conclusion

Our findings indicate that intra-individual variations in voice variables do not significantly differ between individuals with and without PD. This suggests that intra-individual voice variability may not serve as a distinguishing factor for PD diagnosis. Future research should explore alternative methods to assess intra-individual voice variability and its potential role in PD diagnostics.

## Introduction

Voice changes affect 70 to 90% of individuals with Parkinson’s disease (PD) [1]. The progression of PD voice changes inevitably worsens, leading to severe voice impairment in the late stages of the disease that negatively impacts communication and family relationships [2]. Individuals with PD are likely to experience deterioration in intelligibility, perceived in terms of mumbling, tight mouth, slow speech, and loss of clarity [3]. These progressive changes in communication are variable, demoralizing, and bothersome [3]. Difficulty finding words and formulating ideas, along with distractibility, make it challenging for individuals with PD to participate in conversations [3]. Individuals with PD reported that their inability to find words and formulate ideas led them to feel frustrated, depressed, and annoyed. Even when engaged in a conversation, individuals with PD reported that listeners often spoke over them, talked for them, did not wait for an answer, or ignored them [3]. These communication issues are associated with a decreased quality of life in individuals with PD [3]. Considering the impact of voice changes on quality of life, early detection of voice changes is essential to implement evidence-based intervention strategies.

Voice and speech changes are one of the first symptoms of PD and can occur even five years before diagnosis [4]. Currently, the standardized clinical voice test in PD is based on a qualitative evaluation of a specific subitem on the Unified Parkinson’s Disease Rating Scale (UPDRS) [2]. The UPDRS relies on voice change recognition, which is often challenging for the clinical ear to detect. These changes are associated with low volume, high pitch, tremulousness, breathiness, hoarseness, and the inability to maintain loudness spontaneously [5]. Voice changes occur across three domains: interconnected phonation, articulation, and prosody [6]. Phonation involves reduced voice volume, known as hypophonia, and altered voice quality, known as dysphonia [2]. Articulation involves a reduced range of articulatory movements, known as hypokinetic articulation [2]. Prosody involves flattened pitch inflection, known as mono-pitch, and stress loss, known as mono-loudness [2]. Other changes include festination and hesitation of speech [2]. These voice changes in PD are caused by poor activation and coordination of the speech-production muscles. The stiffness and tremor of the larynx muscle hardens the vocal cords, affecting the vibration of the vocal cords and causing voice and speech changes. Additionally, individuals with PD have reduced control of the diaphragm, tongue, and lip muscles [7].

To assess changes in voice and speech, it has been suggested that voice variables could be used for the early detection of PD symptoms [8]. Voice variables consistent with a diagnosis of PD include increased shimmer, increased jitter, reduced harmonics-to-noise ratio (HNR), and changes in fundamental frequency [2]. Despite the expected changes, studies have identified inter-individual voice variations as PD progresses [8]. Beyond the inter-individual voice variations, there is limited research investigating the intra-individual voice variations. Research has only focused on a single voice measurement for individuals with or without PD [9]. The emphasis on only one measurement provides limited information and the potential for an unreliable voice assessment. For example, the individual whose voice is being assessed may be stressed or nervous. Stress and nervousness can be reflected in voice assessments, leading to unreliable findings [10]. Research investigating intra-individual voice variations is needed to assess the reliability of a single voice measurement and potentially make decisions regarding the voice assessment findings. This study aimed to determine whether intra-individual voice variability differs between individuals with and without PD. Our study will provide insight into the use of intra-individual voice variations to identify individuals with and without PD. The secondary purpose was to assess the relationship of single voice measurements with intra-individual voice variations in individuals with and without PD. This study examined whether single voice measurements are sufficient or if repeated measurements provide more reliable insights.

## Materials and methods

This study utilized an existing dataset and was deemed exempt by the Institutional Review Board at the University of Jamestown.

Study design and data source* * 

We utilized the "Oxford Parkinson’s Disease Detection Dataset," which consists of biomedical voice measurements for individuals with and without PD. Each individual's voice was measured six or seven times [11,12]. The dataset provided an opportunity to investigate the intra-individual variations in voice variables. The voice variables used in the study assessment were average, maximum, and minimum vocal fundamental frequency, measures of variation in fundamental frequency, measures of variation in amplitude, measures of the ratio of noise to tonal components, nonlinear dynamical complexity measures, signal fractal scaling exponent, and nonlinear measures of fundamental frequency variation [11].

Study population

The dataset consisted of biomedical voice measurements from individuals with and without PD. The dataset included 22 voice variables, which were grouped into four categories for our study. The categories included (1) vocal fundamental frequency, (2) jitter, (3) shimmer, and (4) other variables. The vocal fundamental frequency variables included Fo, Fhi, and Flo. The jitter variables included Jitter(%), Jitter(Abs), relative average perturbation (RAP), pitch perturbation quotient (PPQ), and difference of differences of periods (DDP). The shimmer variables included Shimmer, Shimmer(dB), APQ3, APQ5, APQ, and difference of differences of amplitudes (DDA). The other variables included noise-to-harmonics ratio (NHR), HNR, recurrence period density entropy (RPDE), correlation dimension (D2), detrended fluctuation analysis (DFA), spread1, spread2, and pitch period entropy (PPE). Voice variable descriptions are available in Table 1. For background information on voice variables, we report the normative ranges for voice variables, and when they could indicate PD. The normative Fo range is 85 to 155 Hz for adult males and 165 to 255 Hz for adult females. The normative Fhi range is 150 to 350 Hz for adult males and 250 to 700 Hz for adult females. The normative Flo range is 75 to 85 Hz for adult males and 135 to 165 Hz for adult females. This normative Fo range includes two to three octaves; a reduction below one octave may indicate PD [13]. The normative ranges for Jitter variables are Jitter(%) ≤ 1.0%, Jitter(Abs) ≤ 0.04 ms, RAP ≤ 0.68%, PPQ ≤ 0.70%, and DDP ≤ 1.36%. Values above the normative ranges for Jitter may indicate PD [13]. The normative ranges for Shimmer variables are Shimmer(%) ≤ 3.5%, Shimmer(dB) ≤ 0.35 dB, APQ3 ≤ 3.5%, APQ5 ≤ 4.5%, APQ ≤ 5.0%, and DDA ≤ 7.0%. Values above the normative ranges for Shimmer may indicate PD [13]. The normative ranges for other voice variables are NHR ≤ 0.2, HNR ≥ 20 dB, RPDE ≤ 0.45, D2 ≤ 3.0, DFA 0.6 to 1.0, spread1 -5 to -3, spread2 0.5 to 2.5, and PPE ≤ 0.6. High NHR or low HNR, higher RPDE and D2, out-of-range DFA, spread1, and spread2, as well as higher PPE, may indicate PD [13].

**Table 1:** Codebook.

Variable	Description	Value labels/codes
Name	ASCII subject name and recording number	-
Fundamental frequency		
Fo	Average vocal fundamental frequency	-
Fhi	Maximum vocal fundamental frequency	-
Flo	Minimum vocal fundamental frequency	-
Jitter		
Jitter(%)	Jitter (percentage)	-
Jitter(Abs)	Jitter (absolute value)	-
RAP	Relative amplitude perturbation	-
PPQ	5-point period perturbation quotient	-
DDP	Average absolute difference of differences between jitter cycles	-
Shimmer		
Shimmer	Shimmer (original unit)	-
Shimmer(dB)	Shimmer (logarithmic unit)	-
APQ3	3-point shimmer perturbation quotient	-
APQ5	5-point shimmer perturbation quotient	-
APQ	11-point amplitude perturbation quotient	-
DDA	Average absolute differences between the amplitudes of consecutive periods	-
Other		
NHR	Noise-to-harmonics ratio of acoustic signals	-
HNR	Harmonics-to-noise ratio of acoustic signals	-
Status	Health status of the subject	0 → no PD 1 → PD
RPDE	Recurrence period density entropy	-
D2	Correlation dimension	-
DFA	Detrended fluctuation analysis	-
Spread1	Nonlinear measure of fundamental frequency variation	-
Spread2	Nonlinear measure of fundamental frequency variation	-
PPE	Pitch period entropy	-

Data analysis

The data were analyzed using STATA 18 (StataCorp LLC Stata statistical software: release 18. College Station, TX: StataCorp LLC. 2023). Descriptive statistics were reported for the voice measurements, including measures of central tendency and dispersion. The changes between each voice measurement for each individual were determined. The averages of these changes were calculated.

Independent samples t-tests were performed to determine significant differences in intra-individual variations in voice variables between individuals with and without PD. Cohen’s d effect size was calculated. The effect sizes were interpreted as trivial effect size (d < 0.20), small effect size (d = 0.20 - 0.49), medium effect size (d = 0.50 - 0.79), large effect size (d = 0.80 - 1.30), and very large effect size (d > 1.30) [14]. The assumption of normality was assessed by generating histograms for each group. The assumption of homogeneity of variance (HOV) was assessed by calculating the variance of each variable to assess the ratio of the larger variance to the smaller variance.

Pearson-product moment correlation coefficients (r) were used to assess the relationship between a single measurement and the average changes. The interpretation of Pearson's r was categorized as weak (< 0.4), moderate (0.4-0.7), and strong (> 0.7) [15]. A p-value of less than 0.05 was considered significant for all analyses.

## Results

The dataset included 32 individuals, of which 24 were diagnosed with PD and eight were without PD. The descriptive statistics for each voice variable are available in Table 2. The means of the variables ranged from -5.68e6 (spread 1) to 1.97e7 (Fhi).

**Table 2:** Descriptive statistics. Sample size n = 32 and observations = 195. PD: Parkinson's Disease; HNR: Harmonics-to-Noise Ratio; Fo: Fundamental Frequency; Fhi: Highest Fundamental Frequency; Flo: Lowest Fundamental Frequency; RAP: Relative Average Perturbation; PPQ: Pitch Perturbation Quotient; DDP: Difference of Differences of Periods; APQ3: Amplitude Perturbation Quotient 3; APQ5: Amplitude Perturbation Quotient 5; APQ: Amplitude Perturbation Quotient; DDA: Difference of Differences of Amplitudes; NHR: Noise-to-Harmonics Ratio; RPDE: Recurrence Period Density Entropy; D2: Correlation Dimension; DFA: Detrended Fluctuation Analysis; PPE: Pitch Period Entropy.

Variable	Mean	SD	Minimum	Maximum
Fundamental frequency				
Fo	1.54e7	4.14e6	8.83e6	2.60e7
Fhi	1.97e7	9.15e6	1.02e7	5.92e7
Flo	1.17	4.35e6	6.55e6	2.39e7
Jitter				
Jitter(%)	6.22e-3	4.85e-3	1.68e-3	3.32e-2
Jitter(Abs)	4.4e-5	3.50e-5	7.00e-6	2.60e-4
RAP	3.31e-3	2.97e-3	6.80e-4	2.14e-2
PPQ	3.45e-3	2.76e-3	9.20e-4	1.96e-2
DDP	9.92e-3	8.90e-3	2.04e-3	6.43e-2
Shimmer				
Shimmer	2.97e-2	1.89e-2	9.54e-3	1.19e-1
Shimmer(dB)	1.19e3	1.18e4	8.50e-2	1.30e5
APQ3	1.57e-2	1.02e-2	4.55e-3	5.65e-2
APQ5	1.79e-2	1.20e-2	5.70e-3	7.94e-2
APQ	2.41e-2	1.69e-2	7.19e-3	1.38e-1
DDA	4.70e-2	3.05e-2	1.36e-2	1.69e-1
Other				
NHR	2.48e-2	4.04e-2	6.50e-4	3.15e-1
HNR	2.19e6	4.43e5	8.44e5	3.30e6
RPDE	4.99e-1	1.04e-1	2.57e-1	6.85e-1
D2	2.38e6	3.83e5	1.42e6	3.67e6
DFA	7.18e-1	5.53e-2	5.74e-1	8.25e-1
Spread1	-5.68e6	1.09e6	-7.96e6	-2.43e6
Spread2	2.27e-1	8.34e-2	6.27e-3	4.50e-1
PPE	2.07e-1	9.01e-2	4.45e-2	5.27e-1

Assumptions

The assumption of normality appeared reasonable for all variables. The assumption of homogeneity of variance appeared reasonable for HNR (ratio 1.09), DFA (ratio 2.23), spread1 (ratio 2.61), spread2 (ratio 2.59), and D2 (ratio 1.37). For all other variables, the assumption of homogeneity of variance was not reasonable, as the ratio of larger to smaller variance was greater than three [16]. Despite the small sample size in individuals without PD and violations of the HOV, the independent samples t-test was still used for data analysis. Evidence indicates that the t-test is a robust test, especially if the assumption of normality is reasonable. Alternative non-parametric tests were considered but may be less powerful than a t-test in detecting differences between groups [17].

Fundamental frequency

The independent samples t-tests revealed no statistically significant differences in intra-individual variations between individuals with and without PD for any of the vocal fundamental frequency variables. The mean differences for these variables ranged from -1.98e5 (Flo) to 2.58e5 (Fhi), with effect sizes ranging from trivial to small (Table 3).

**Table 3:** Results of the independent samples t-tests. PD: Parkinson's Disease; HNR: Harmonics-to-Noise Ratio; Fo: Fundamental Frequency; Fhi: Highest Fundamental Frequency; Flo: Lowest Fundamental Frequency; RAP: Relative Average Perturbation; PPQ: Pitch Perturbation Quotient; DDP: Difference of Differences of Periods; APQ3: Amplitude Perturbation Quotient 3; APQ5: Amplitude Perturbation Quotient 5; APQ: Amplitude Perturbation Quotient; DDA: Difference of Differences of Amplitudes; NHR: Noise-to-Harmonics Ratio; RPDE: Recurrence Period Density Entropy; D2: Correlation Dimension; DFA: Detrended Fluctuation Analysis; PPE: Pitch Period Entropy.

Variable	MD	95% CI	P-value	Cohen’s d
Fundamental frequency				
Fo	-3.39e4	-2.99e5, 2.31e5	0.80	-0.11 (trivial)
Fhi	2.58e5	-1.33e6, 1.84e6	0.74	0.14 (trivial)
Flo	-1.98e5	-7.56e5, 3.59e5	0.47	-0.30 (small)
Jitter				
Jitter(%)	2.70e-4	-3.91e-4, 9.32e-4	0.41	0.34 (small)
Jitter(Abs)	1.05e-6	-3.59e-6, 5.68e-6	0.65	0.19 (trivial)
RAP	1.77e-4	-2.28e-4, 5.82e-4	0.38	0.36 (small)
PPQ	1.85e-4	-2.01e-4, 5.70e-4	0.34	0.40 (small)
DDP	5.32e-4	-6.84e-4, 1.75e-3	0.38	0.36 (small)
Shimmer				
Shimmer	4.28e-4	-2.26e-3, 3.11e-3	0.75	0.13 (trivial)
Shimmer(dB)	7.07e2	-1.82e3, 3.24e3	0.57	0.23 (small)
APQ3	2.88e-4	-1.27e-3, 1.84e-3	0.71	0.15 (trivial)
APQ5	2.11e-4	-1.34e-3, 1.76e-3	0.78	0.11 (trivial)
APQ	2.69e-4	-1.59e-3, 2.13e-3	0.77	0.12 (trivial)
DDA	8.64e-4	-3.80e-3, 5.52e-3	0.71	0.15 (trivial)
Other				
NHR	2.03e-3	-4.00e-3, 8.06e-3	0.50	0.28 (small)
HNR	-2.76e4	-6.86e4, 1.34e4	0.18	-0.56 (medium)
RPDE	9.09e-4	-1.69e-2, 1.51e-2	0.91	-0.05 (trivial)
D2	1.33e4	-5.47e4, 8.13e4	0.69	0.16 (trivial)
DFA	-3.99e-3	-9.42e-3, 1.43e-3	0.14	-0.61 (medium)
spread1	6.02e4	-1.09e5, 2.29e5	0.47	0.30 (small)
spread2	3.90e-4	-1.53e-2, 1.61e-2	0.96	0.02 (trivial)
PPE	5.59e-3	-7.69e-3, 1.89e-2	0.40	0.35 (small)

There was a weak to strong relationship between individual voice measurements and the average intra-individual voice variations. Fo showed a weak to strong negative correlation (-0.30 to -0.80), Fhi showed a weak to moderate positive correlation (0.10 to 0.46), and Flo showed a weak to strong negative correlation (0.02 to -0.83). The strongest correlation was at measurement 7 for Flo (r = -0.83) (Table 4).

**Table 4:** Results of the Pearson's correlations. * p < 0.05; ** p <0.01 PD: Parkinson's Disease; HNR: Harmonics-to-Noise Ratio; Fo: Fundamental Frequency; Fhi: Highest Fundamental Frequency; Flo: Lowest Fundamental Frequency; RAP: Relative Average Perturbation; PPQ: Pitch Perturbation Quotient; DDP: Difference of Differences of Periods; APQ3: Amplitude Perturbation Quotient 3; APQ5: Amplitude Perturbation Quotient 5; APQ: Amplitude Perturbation Quotient; DDA: Difference of Differences of Amplitudes; NHR: Noise-to-Harmonics Ratio; RPDE: Recurrence Period Density Entropy; D2: Correlation Dimension; DFA: Detrended Fluctuation Analysis; PPE: Pitch Period Entropy.

Variable	1	2	3	4	5	6	7
Fundamental frequency (average change)							
Fo	-0.30	-0.51**	-0.47**	-0.51**	-0.50**	-0.56**	-0.80
Fhi	0.46**	0.34	0.43*	0.24	0.10	-0.42*	-0.29
Flo	0.30	-0.39*	-0.36*	0.02	-0.34	-0.54**	-0.83
Jitter (average change)							
Jitter(%)	-0.54**	-0.58**	-0.76**	-0.83**	-0.72**	-0.89**	-0.99
Jitter(abs)	-0.43*	-0.46**	-0.69**	-0.82**	-0.63**	-0.85**	-0.96
RAP	-0.59**	-0.62**	-0.80**	-0.87**	-0.72**	-0.90**	-1.00*
PPQ	-0.54**	-0.53**	-0.75**	-0.84**	-0.77**	-0.89**	-1.00*
DDP	-0.59**	-0.62**	-0.80**	-0.87**	-0.72**	-0.90**	-1.00*
Shimmer (average change)							
Shimmer	0.31	0.02	-0.12	-0.40*	-0.26	-0.43*	-0.69
Shimmer(dB)	-0.55**	-0.41*	-0.49**	-0.51**	-0.52**	-1.00**	-1.00**
APQ3	0.29	-0.01	-0.17	-0.47**	-0.26	-0.52**	-0.84
APQ5	0.37*	0.06	0.01	-0.25	-0.21	-0.30	-0.47
APQ	0.39*	0.15	0.06	-0.14	-0.16	-0.14	0.08
DDA	0.29	-0.01	-0.17	-0.47**	-0.26	-0.52**	-0.84
Other (average change)							
NHR	-0.57**	-0.76**	-0.90**	-0.89**	-0.70**	-0.91**	-0.99
HNR	-0.16	-0.18	-0.45**	-0.48**	-0.55**	-0.62**	-1.00**
RPDE	0.34	0.06	-0.02	-0.02	-0.18	-0.49**	-0.88
D2	0.48**	0.10	0.24	0.05	0.07	-0.52**	-0.29
DFA	-0.22	-0.42*	-0.50**	-0.64**	-0.51**	-0.68**	0.48
spread1	0.12	-0.01	-0.03	-0.15	-0.19	-0.65**	-0.22
spread2	0.33	0.13	-0.05	-0.03	-0.12	-0.60**	-0.02
PPE	0.16	0.01	0.00	-0.20	-0.26	-0.57**	0.15

Jitter

The independent samples t-tests revealed no statistically significant differences in intra-individual variations between individuals with and without PD for any of the jitter variables. The mean differences for these variables ranged from 1.05e-6 (Jitter(Abs)) to 5.32e-4 (DDP), with effect sizes ranging from trivial to small (Table 3).

There was a moderate to strong relationship between individual voice measurements and the average intra-individual voice variations. Jitter(%) showed a moderate to strong negative correlation (-0.54 to -0.99), Jitter(abs) showed a moderate to strong negative correlation (-0.43 to -0.96), and RAP showed a moderate to strong negative correlation (-0.59 to -1.00). Similarly, PPQ and DDP showed a moderate to strong negative correlation (-0.54 to -1.00). The strongest correlation was at measurement 7 for RAP, PPQ, and DDP (r = -1.00) (Table 4).

Shimmer

The independent samples t-tests revealed no statistically significant differences in intra-individual variations between individuals with and without PD for any of the shimmer variables. The mean differences for these variables ranged from 2.11e-4 (APQ5) to 7.07e2 (Shimmer(dB)), with effect sizes ranging from trivial to small (Table 3).

There was a weak to strong relationship between individual voice measurements and the average intra-individual voice variations. Shimmer showed a weak to moderate negative correlation (-0.12 to -0.69), and Shimmer(dB) showed a moderate to strong negative correlation (-0.41 to -1.00). APQ3 showed a weak to moderate negative correlation (-0.01 to -0.84), while APQ5 showed a weak to moderate negative correlation (-0.25 to -0.47). APQ showed a weak negative to weak positive correlation (-0.14 to 0.39), and DDA showed a weak to moderate negative correlation (-0.01 to -0.84). The strongest correlation was at measurement 7 for Shimmer(dB) (r = -1.00) (Table 4).

Other voice variables

The independent samples t-tests revealed no statistically significant differences in intra-individual variations between individuals with and without PD for any of the other voice variables. The mean differences for these variables ranged from -2.76e6 (HNR) to 6.02e4 (spread1), with effect sizes ranging from trivial to medium (Table 3).

There was a weak to strong relationship between other voice measurements and the average intra-individual voice variations. NHR showed a moderate to strong negative correlation (-0.57 to -0.99), while HNR showed a weak to strong negative correlation (-0.16 to -1.00). RPDE showed a weak to strong negative correlation (-0.02 to -0.88), and D2 showed a weak to moderate positive correlation (0.05 to 0.48). DFA showed a weak to moderate negative correlation (-0.22 to -0.68), while spread1 and spread2 showed weak negative to weak positive correlations (-0.15 to 0.33). PPE showed a weak positive correlation (0.00 to 0.16). The strongest correlation was at measurement 7 for HNR (r = -1.00) (Table 4).

## Discussion

Our study aimed to examine intra-individual variations in voice between individuals with and without PD. We found that voice variables independently had a trivial to medium effect that was not significantly different between individuals with and without PD. This suggests that intra-individual voice variability may not serve as a distinguishing factor for PD diagnosis. Despite the lack of significant findings, the study provides important insights into the intra-individual variability of voice in PD and highlights the need for further research to understand the relationship between PD, voice variability, and other influencing factors.

Intra-individual voice variations

To our knowledge, we are the first to examine intra-individual variations in voice between individuals with and without PD. We observed weak to strong correlations between individual voice measurements and average intra-individual voice variations. The strongest correlations were observed at the final measurement for several variables, including RAP, PPQ, DDP, shimmer (dB), and HNR. These findings suggest that while group differences were not significant, there are meaningful intra-individual fluctuations in voice variables with repeated practice, particularly within specific measurement points. This could also suggest that individuals might improve with practice, as repeated voice assessments may enhance vocal control or reduce stress-related variability. Our findings are in contrast to others who reported that individuals with PD exhibited significantly greater intra-individual variations in voice, which was linked to disease progression. Variations in vocal loudness and pitch were found to be more pronounced in individuals with PD compared to those without PD, and fluctuations in speech patterns were associated with motor symptoms of the disease [18]. This contrast suggests that intra-individual voice variability may become more pronounced over time as PD progresses. In the short term, factors such as stress levels and day-to-day fluctuations may be comparable between individuals with and without PD, potentially masking potential differences in our investigation. Our findings underscore the importance of longitudinal assessments to capture the progressive nature of voice variability in PD.

Inter-individual voice variations

Inter-individual voice variations are the variations that occur across individuals. Intra-individual variability can influence the degree of inter-individual differences. High variability within individuals may contribute to increased variability across individuals, potentially affecting the reliability of voice assessments. A systematic review and meta-analysis found that some voice variables (Jitter(%), Shimmer, Shimmer(dB), Fo) had significant variations between individuals with and without PD [19]. In contrast to our study, which examined voice variations within the same individual, this systematic review and meta-analysis examined the variations across individuals. While our primary focus was on intra-individual variations, the SDs of voice variables across participants in our study offer additional insights into inter-individual variability. For example, the SDs for key variables such as Jitter (%), Shimmer, and Fo indicate notable variability across individuals with and without PD.

Potential influential factors

Beyond a diagnosis of PD, there are a range of influential factors that may affect voice measurements. Our findings, which showed that intra-individual variations in all voice variables did not significantly differ between individuals with and without PD, suggest that other influential factors should be considered when interpreting voice measurements.

Fatigue is particularly relevant in PD. However, fatigue can also impact individuals without PD, particularly older adults. Increased physical and cognitive fatigue in aging populations may contribute to voice instability, potentially confounding differences between individuals with and without PD. Individuals with PD often experience increased physical and cognitive fatigue, which could impact voice stability [20]. Previous research has indicated that vocal fatigue can lead to increased jitter and shimmer, as well as reduced vocal intensity and endurance [21]. Similarly, medication effects must be considered, as dopaminergic treatments can influence vocal control [22]. Some studies suggest that while medication can improve speech loudness for those with PD, it may not consistently affect measures of intra-individual variability in voice [23]. The timing of medication intake relative to voice assessments may also contribute to variability in findings. Among these factors, stress is known to influence vocal parameters. For instance, experimentally induced stress increases vocal fundamental frequency, jitter, and shimmer relative to baseline [10]. However, it remains unclear whether intra-individual variability in voice is sensitive to stress. These findings highlight the need for further research to understand the relationship between voice variability, PD, and potential influential factors. Prior to conducting voice measurements, clinicians should consider assessing for the presence of other influential factors. Future research should investigate whether stress-induced voice changes exhibit different patterns in individuals with and without PD and how these changes contribute to intra-individual variability. Additionally, studies should assess whether stress management strategies or relaxation techniques could stabilize intra-individual voice variations in both populations.

Study limitations

The study included a relatively small sample size, particularly within the control group, which consisted of only eight individuals without PD. Small sample sizes can limit the statistical power of analyses, making it difficult to detect significant differences even if they exist [24].

The variability observed in voice measurements may also be influenced by the type and difficulty of the voice task used. Task complexity can introduce variability in voice performance, even in individuals without PD. Tasks that require prolonged concentration may lead to greater voice variability due to psychological fatigue or stress, which could mask differences between individuals with PD and individuals without PD [10].

Future research

Future research should focus on conducting longitudinal assessments to track intra-individual voice variability over time, as this may provide better insights into disease progression. Additionally, investigating whether repeated voice assessments improve vocal stability in individuals with PD could help determine whether practice effects play a role in voice variability. Larger and more balanced groups for individuals with and without PD should be included to increase statistical power and strengthen findings. Furthermore, research should explore how external factors such as stress, fatigue, and medication influence intra-individual voice variability and whether these factors contribute differently to voice stability in individuals with PD compared to healthy controls.

## Conclusions

Our findings indicate that intra-individual variations in voice variables do not significantly differ between individuals with and without PD. This suggests that intra-individual voice variability may not serve as a distinguishing factor for PD diagnosis. Future research should not only focus on alternative methods for measuring variability but also on identifying the best approach for integrating voice analysis into clinical assessments. It should explore alternative methods to assess intra-individual voice variability and its potential role in PD diagnostics and determine the best approach for integrating voice analysis into clinical assessments. The complexity of PD-related voice changes, together with the influence of task-related factors, highlights the need for further research into the relationships between voice variability, PD, and other influential factors.

## References

[1] Logemann JA, Fisher HB, Boshes B, Blonsky ER (1978). Frequency and cooccurrence of vocal tract dysfunctions in the speech of a large sample of Parkinson patients. J Speech Hear Disord.

[2] Ma A, Lau KK, Thyagarajan D (2020). Voice changes in Parkinson's disease: What are they telling us?. J Clin Neurosci.

[3] Miller N, Noble E, Jones D, Burn D (2006). Life with communication changes in Parkinson's disease. Age Ageing.

[4] Harel B, Cannizzaro M, Snyder PJ (2004). Variability in fundamental frequency during speech in prodromal and incipient Parkinson's disease: a longitudinal case study. Brain Cogn.

[5] Liotti M, Ramig LO, Vogel D (2003). Hypophonia in Parkinson's disease: neural correlates of voice treatment revealed by PET. Neurology.

[6] Critchley EM (1981). Speech disorders of Parkinsonism: a review. J Neurol Neurosurg Psychiatry.

[7] Yang S, Wang F, Yang L (2020). The physical significance of acoustic parameters and its clinical significance of dysarthria in Parkinson's disease. Sci Rep.

[8] Convey RB, Laukkanen AM, Ylinen S, Penttilä N (2024). Analysis of voice changes in early-stage Parkinson’s disease with AVQI and ABI: a follow-up study. J Voice.

[9] Suppa A, Costantini G, Asci F (2022). Voice in Parkinson's disease: a machine learning study. Front Neurol.

[10] Mendoza E, Carballo G (1998). Acoustic analysis of induced vocal stress by means of cognitive workload tasks. J Voice.

[11] Little MA, McSharry PE, Hunter EJ, Ramig LO (2008). Suitability of dysphonia measurements for telemonitoring of Parkinson's disease. IEEE Trans Biomed Eng.

[12] Little MA, McSharry PE, Roberts SJ, Costello DA, Moroz IM (2007). Exploiting nonlinear recurrence and fractal scaling properties for voice disorder detection. Biomed Eng Online.

[13] Baken RJ, Orlikoff RF (2000). Clinical Measurement of Speech and Voice.

[14] Maher JM, Markey JC, Ebert-May D (2013). The other half of the story: effect size analysis in quantitative research. CBE Life Sci Educ.

[15] Cohen J (1988). Statistical Power Analysis for the Behavioral Sciences.

[16] Fávero LP, Belfiore P, de Freitas Souza R (2023). Hypotheses tests. Data Science, Analytics and Machine Learning with R.

[17] Field A (2013). Discovering Statistics Using IBM SPSS Statistics. http://repo.darmajaya.ac.id/5678/1/Discovering%20Statistics%20Using%20IBM%20SPSS%20Statistics%20%28%20PDFDrive%20%29.pdf.

[18] Miller N (2017). Communication changes in Parkinson's disease. Pract Neurol.

[19] Chiaramonte R, Bonfiglio M (2020). Acoustic analysis of voice in Parkinson's disease: a systematic review of voice disability and meta-analysis of studies. Rev Neurol.

[20] Kostić VS, Tomić A, Ječmenica-Lukić M (2016). The pathophysiology of fatigue in Parkinson’s disease and its pragmatic management. Mov Disord Clin Pract.

[21] Lei Z, Fasanella L, Martignetti L, Li-Jessen NY, Mongeau L (2020). Investigation of vocal fatigue using a dose-based vocal loading task. Appl Sci (Basel).

[22] Cavallieri F, Budriesi C, Gessani A (2020). Dopaminergic treatment effects on dysarthric speech: Acoustic analysis in a cohort of patients with advanced Parkinson’s disease. Front Neurol.

[23] Tykalova T, Novotny M, Ruzicka E, Dusek P, Rusz J (2022). Short-term effect of dopaminergic medication on speech in early-stage Parkinson's disease. NPJ Parkinsons Dis.

[24] Button KS, Ioannidis JP, Mokrysz C, Nosek BA, Flint J, Robinson ES, Munafò MR (2013). Power failure: why small sample size undermines the reliability of neuroscience. Nat Rev Neurosci.

